# Fifth-time redo mitral valve replacement via right thoracotomy under systemic hyperkalemia cardiopulmonary bypass without aortic cross-clamp

**DOI:** 10.1051/ject/2023040

**Published:** 2023-12-15

**Authors:** Tomohisa Takeichi, Yoshihisa Morimoto, Akitoshi Yamada, Takanori Tanaka

**Affiliations:** 1 Department of Clinical Engineering, Kitaharima Medical Center 926-250, Ichiba-cho Ono-shi Hyogo 675-1392 Japan; 2 Department of Cardiovascular Surgery, Kitaharima Medical Center 926-250, Ichiba-cho Ono-shi Hyogo 675-1392 Japan

**Keywords:** Cardiopulmonary bypass (CPB), Systemic hyperkalemia, Right thoracotomy, Reoperation

## Abstract

The surgical management of prosthetic valvular endocarditis (PVE) can be challenging. We report a case of a 46-year-old female patient who had a history of four cardiac operations. We chose a mitral valve replacement via right thoracotomy to enable optimal exposure of the mitral valve (MV). Because of multi-reoperations, we employed systemic hyperkalemia for cardiac arrest to protect the heart during cardiopulmonary bypass (CPB) without aortic cross-clamping. Here, we present a complex operation that performed management of CPB under hyperkalemia and the patient had a good postoperative recovery.

## Introduction

Five times of cardiac reoperations for PVE are a complex operation with significant risk. Due to the difficulty of aortic cross-clamping, mitral valve replacement (MVR) is performed under beating heart (BH) or ventricular fibrillation (VF) conditions. On-pump BH or VF valvular operations may have some technical advantages and utilities [1]. However, some studies have shown that the BH alternative with the VF approach is inferior to the empty heart technique due to its reduction in oxygen delivery to the subendocardium and the consequent suboptimal myocardial protection [2, 3]. In this case, we determined that it was difficult to perform MVR via the right thoracotomy procedure under the BH and VF techniques. Because, we had no previous experience with the BH technique in complex reoperation cases and, there was a need to secure a surgical field of view close to cardiac arrest. Moreover, though the VF condition was not an inappropriate myocardial protection strategy [4], in this case, the prolonged operative time was anticipated. The longer the VF time, the more reduction in oxygen delivery to the subendocardium, and the more creatine-kinase MB (CK-MB) increases [5, 6]. Therefore, in the case of VF, we thought that weaning from CPB was difficult under the prolonged VF time, and we chose the systemic hyperkalemia strategy during CPB to protect the heart.

This study was approved by the Institutional Review Board at Kitaharima Medical Center (IRB-0443) with the waiver of informed consent.

## Case report

The patient (height 156 cm; weight 41 kg) had a history of recurrent cardiac operations, starting with a MVR at the age of 31 for infective endocarditis. At the age of 33, she underwent two further reoperations, including MVR for a stuck valve and patch repair for pseudoaneurysm of the ascending aorta due to mediastinitis. Additionally, an omental wrapping was performed. At the age of 42, the patient underwent an emergency MVR via right thoracotomy under VF of the stuck valve.

**Figure 1:**
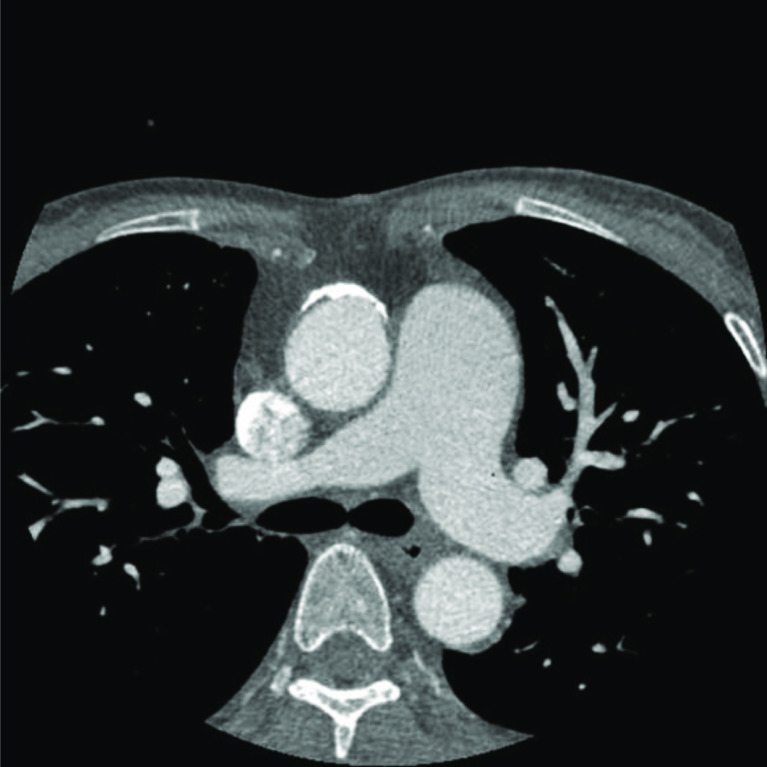
Preoperative contrast-CT indicates patch repair for pseudoaneurysm of the ascending aorta. CT: Computed Tomography.

**Figure 2:**
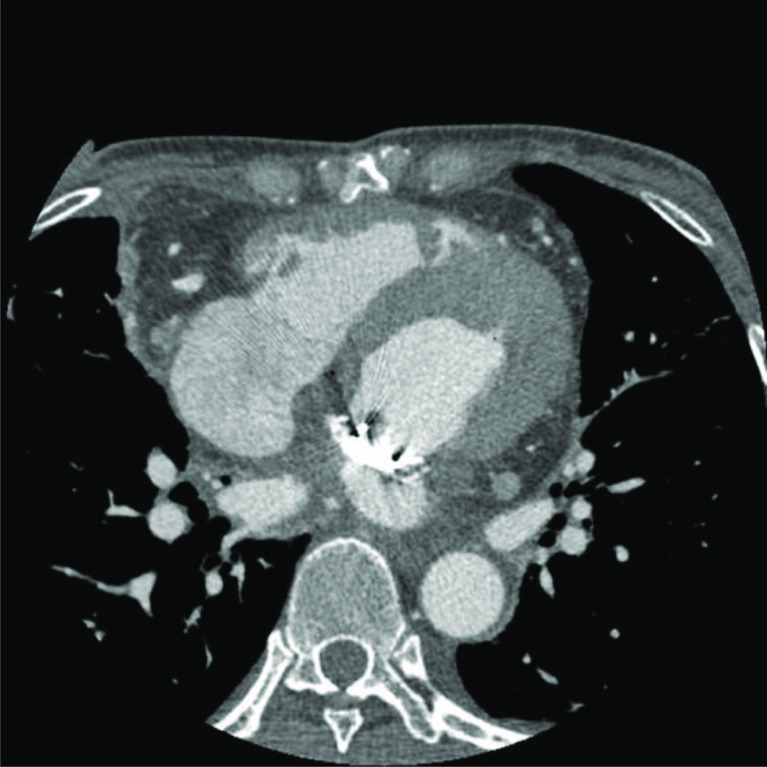
Preoperative contrast CT indicates that omental tissue covers from the right atrium to the entire surface of the aorta. CT: Computed Tomography.

This time, the patient was diagnosed with sepsis and prosthetic valve endocarditis (PVE) by transesophageal echocardiography (TEE) and positive blood cultures in our clinical microbiology laboratory. Mitral valve and aortic valve regurgitation were not confirmed. A five times MVR via right thoracotomy procedure was planned. We performed systemic hyperkalemia for cardiac arrest during CPB without aortic cross-clamping because of computed tomography (CT) scan showed that omental tissue covered from the right atrium to the entire surface of the aorta (Figures 1 and 2).

Following induction of general anesthesia, the patient underwent MVR via a right thoracotomy procedure. CPB was established with a venous cannula 22/22Fr (MICS Cannulae; LivaNova, Tokyo, Japan) placed in the right femoral vein and an arterial cannula 16Fr (PCKC-A, MERA, Tokyo, Japan) placed in the right femoral artery. Centrifugal pump (MERA Centrifugal Pump HCF-MP23, SENKO MEDICAL INSTRUMENT, Inc., Tokyo, Japan) was used for CPB, which target pump flow was 2.6 L/min per m^2^. Phenylephrine and noradrenaline were administered to maintain a mean arterial pressure above 70 mmHg. Anticoagulation was given at an initial dose of 300 IU/kg to achieve a goal-activated clotting time of at least 480 s and if activated clotting time was less than 480 s, an additional dose of 4000 IU was given. A CDI Blood Parameter Monitoring System 500 (Terumo, Tokyo, Japan) was recalibrated every 30 min, and an arterial blood gas sample was also checked every 30 min. The patient was cooled to 28 °C centigrade. To perform myocardial protection due to systemic hyperkalemia, we used 10 mL of MgSO_4_ and a mixture solution that mixed 500 mL of bicarbonate ringer solution with 50 mL of KCL 10 mEq/L.

**Figure 3:**
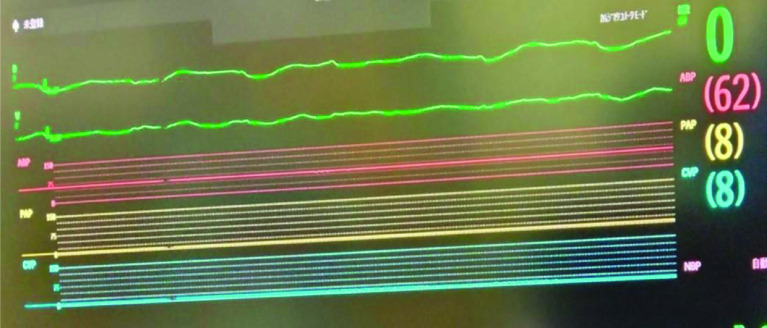
ECG of systemic hyperkalemia during CPB shows a blood potassium level of 8 mEq/L. ECG: Electrocardiogram; CPB: Cardiopulmonary bypass.

**Figure 4:**
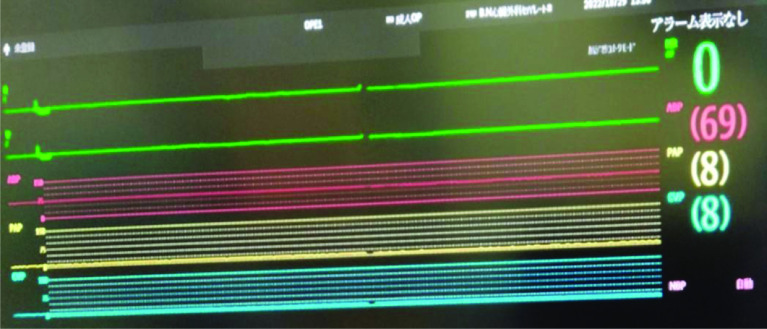
ECG of systemic hyperkalemia during CPB shows a blood potassium level of 9 mEq/L. ECG: Electrocardiogram; CPB: Cardiopulmonary bypass.

When VF occurred at 28.0°, the left atrium opened via right thoracotomy. We administered an initial dose of 10 mL of MgSO_4_ and 500 mL of mixture solution to achieve cardiac arrest. Immediately following the administration, VF stopped and the duration was 3 min. To maintain cardiac arrest, we continuously infused a solution to maintain a blood potassium level of 9 mEq/L and administered 10 mL of MgSO_4_ every 30 min. Because it was not possible to obtain cardiac arrest at a blood potassium concentration of 8.0 mEq/L (Figures 3 and 4). The body temperature was maintained at 28.0°. After thorough debridement of the extensive vegetation and removal of the artificial valve, redo MVR was performed using Epic 25 mm (Abbott Medical) valve. When a left ventricular vent through the MV was useful in preventing aortic valve release, we started rewarming the temperature and lowering potassium levels in the blood by using dilutional ultrafiltration (DUF). Also, we used continuous glucose-insulin therapy. To decrease from 9 mEq/L to 5.5 mEq/L level of potassium in the blood, we took about 60 min. The potassium level after CPB weaning was 5.5 mEq/L. The peak potassium level was 9.9 mEq/L, the minimum was 8.0 mEq/L, and the total potassium administered was 250mEq. Weaning from CPB was performed using catecholamines, primarily dobutamine which could easily wean. Cardiac arrest time and CPB time were 180 min and 273 min, respectively.

The patient’s postoperative maximum CK-MB level was 19. The duration of mechanical ventilation and length of stay in the intensive care unit (ICU) was 16 h and 3 days, respectively. The postoperative course was uneventful and she was discharged after undergoing antibiotic treatment for 6 weeks.

Informed consent to report patient information and images was obtained.

## Discussion

Reoperative valve surgery is acknowledged to be more complex and increases morbidity and mortality rates [1]. The techniques of MV reoperation are becoming important because many patients have received MV repair or bioprosthetic valve replacement over the last few decades, and these patients are prone to revisional operations in the future. The right thoracotomy approach for reoperations enables optimal exposure of the MV. Redo MV procedures can be performed on either the empty BH or in case of VF arrest and systemic hyperkalemia arrest [2, 6]. However, some researchers showed that the VF approach is inferior to the empty heart technique due to its reduction in oxygen delivery to the sub-endocardium and the subsequent suboptimal myocardial protection [2, 3]. The flow to the sub-endocardium occurs during diastole. The compressive force exerted on the sub-endocardial muscle by fibrillation restricts the flow and oxygen delivery to the myocardium during the diastolic phase of ventricular fibrillation [7]. Imanaka et al. showed that the more prolonged VF time, CK-MB increased and the longest VF time was 146 min. However, such an extended period of VF did not cause severe myocardial damage [5]. Also, Hiraoka et al. reported that intraoperative myocardial protection under mild hypothermia and VF was not an inappropriate myocardial protection strategy compared to cardiac arrest with a cardioplegic solution [4].

On the other hand, the BH approach and systemic hyperkalemia avoid sub-endocardial hypoperfusion mismatches, which are commonly observed with VF. Additionally, during ischemic arrest, myocardial edema increases in the static diastolic state and may lead to cardiac dysfunction [6]. In the case of systemic hyperkalemia, provides complete electromechanical diastolic arrest with uniform myocardial protection as opposed to fibrillatory arrest without the fear of cardioplegia washout [8, 9]. Some studies have suggested that preoperative cardiac surgery can be safely performed with previous CABG and internal thoracic artery (ITA) using systemic hyperkalemia and arrest without the ITA graft [8–10].

In this case, we employed systemic hyperkalemia for the complex reoperation and were able to safely perform myocardial protection. To achieve myocardial protection, we administered an initial dose of 10 mL of MgSO_4_ and 500 mL of mixture solution and maintained a systemic potassium concentration of 9.0 mEq/L by continuously infusing a drop and administrated 10 mL of MgSO_4_ every 30 min. The peak potassium level was 9.9 mEq/L, the minimum was 8.0 mEq/L, and the total potassium administered was 250 mEq. Weaning from CPB was easy and the maximum level of postoperative CK-MB was 19. Some studies show that cardiac protection due to systemic hyperkalemia without aortic cross-clamp can be a safe option and protect the heart [8, 11]. Moreover, compared to hypothermic cardiac arrest, hyperkalemia cardiac arrest is associated with decreased myocardial adenosine triphosphate level [12]. In our series, CK-MB was slightly increased. In terms of becoming 9.9 mEq/L of potassium level, we calibrated the CDI parameter at 30-minute intervals. However, since the potassium concentration of CDI can only be measured up to 8.0 mEq/L when we checked an arterial blood gas sample every 30 min, the peak potassium level was 9.9 mEq/L. In this case, as it was not possible to obtain cardiac arrest at a blood potassium concentration of 8.0 mEq/L, we maintained a blood potassium level of 9 mEq/L.

However, this method of systemic hyperkalemia to achieve cardiac arrest has some drawbacks. First of all, to maintain a mean arterial pressure above 70 mmHg, we used phenylephrine and noradrenaline. However other reports suggest that systemic hyperkalemia may reduce vascular tone by affecting potassium channels [13, 14]. Next, it may take some time to decrease the systemic potassium concentration. In this case, we used DUF methods and glucose-insulin therapy to return to normal potassium levels. Intracellular potassium is moved to the extracellular compartment during rewarming, suggesting that hyperkalemia is caused by administered potassium as well as by transport from the intracellular compartment and its effective removal is crucially important. In fact, we took about 60 min to decrease the potassium level to 5.5 mEq/L. To prevent postoperative fatal arrhythmia due to hyperkalemia, the most important procedure is to remove excessive serum potassium during CPB [15]. However, it is difficult to decrease high potassium levels in a short time using DUF alone. A study has shown that the use of the gravity drainage type hemodiafiltration method is useful for correcting hyperkalemia occurring during extracorporeal circulation because of its simple structure [16].

We believe this strategy is useful in complex cases to protect the heart. However, some researches show that CPB under systemic hyperkalemia is safe, few studies have been reported on via right thoracotomy under systemic hyperkalemia CPB without aortic cross-clamping. Therefore, this technique warrants further evaluation.

## Data Availability

All available data are incorporated into the article.
